# P-1705. Efficacy of Joint Fluid Cultures Submitted in BD Bactec® Bottles Compared to Routine Culture on Solid Media: A Quality Assurance Validation

**DOI:** 10.1093/ofid/ofaf695.1877

**Published:** 2026-01-11

**Authors:** Thein Myint, Julie A Ribes

**Affiliations:** University of Kentucky, Lexington, Kentucky; University of Kentucky, Lexington, Kentucky

## Abstract

**Background:**

Some institutions utilize blood culture bottles (BCB) as part of their culture for sterile fluids. This practice may increase the rate of culture positivity. When performed as designed, these fluids should be supplemented with sterile blood or nutrients. Our center has not validated the use of these bottles for non-blood samples. Nonetheless, physicians often submit joint samples in BCB, so archival data are available for analysis for this sample source to determine the overall efficacy of culture using BCB compared to standard culture techniques.

Table

**Table 1:**
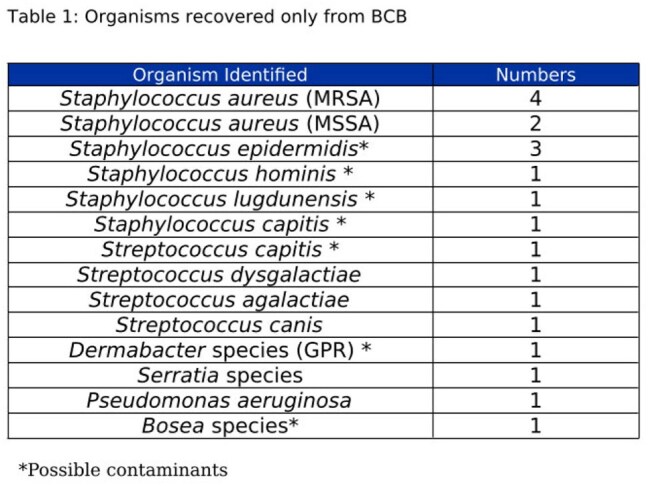
Organisms recovered only from BCB

**Methods:**

Electronical medical record searches were performed for the year 2020 (SUNQUEST) and June 2021- July 2022 (EPIC) to identify all positive cultures for joint fluids produced by BD Bactec® bottles associated with routine cultures for correlation.

**Results:**

From June 2021 to July 2022, 168 blood culture bottles with joint fluid were submitted and 43 (25.6%) were positive.

During the two-year period, there were 79 growth-positive joint BCB with routine culture results. Of these, 59 (74.7%) demonstrated 100% concordance by both culture methods and 20 (25.3%) produced discrepant results (table 1). Three of these discrepant results represented growth of fewer organisms on solid media compared to the bottles and the remaining 17 demonstrated growth only in the BCB. These discrepant cultures represented 6 *Staphylococcus aureus* (2 MRSA), 6 coagulase negative *Staphylococci*, 4 *Streptococcal*species, 1 Gram positive rod, and 3 Gram negative rods.

**Conclusion:**

Adding BCB to the standard culture increases the rate of culture positivity including the contaminants. Seventy-five percent (59/79) of the BCB cultures were duplicative, whereas 25% provided new culture data not seen in the routine culture. Sixty-five percent of discrepant growth (13/20) in BCB were significant whereas the remaining 35% (7/20) would have been classified as probable contaminants by blood cultured standards. This shows that, even without the addition of the recommended supplements, BCB can support bacterial growth from many of these samples.

**Disclosures:**

All Authors: No reported disclosures

